# Experimental Study of the Flexural and Compression Performance of an Innovative Pultruded Glass-Fiber-Reinforced Polymer-Wood Composite Profile

**DOI:** 10.1371/journal.pone.0140893

**Published:** 2015-10-20

**Authors:** Yujun Qi, Wei Xiong, Weiqing Liu, Hai Fang, Weidong Lu

**Affiliations:** College of Civil Engineering, Nanjing Tech University, Nanjing, China; Monash University, AUSTRALIA

## Abstract

The plate of a pultruded fiber-reinforced polymer or fiber-reinforced plastic (FRP) profile produced via a pultrusion process is likely to undergo local buckling and cracking along the fiber direction under an external load. In this study, we constructed a pultruded glass-fiber-reinforced polymer-light wood composite (PGWC) profile to explore its mechanical performance. A rectangular cross-sectional PGWC profile was fabricated with a paulownia wood core, alkali-free glass fiber filaments, and unsaturated phthalate resin. Three-point bending and short column axial compression tests were conducted. Then, the stress calculation for the PGWC profile in the bending and axial compression tests was performed using the Timoshenko beam theory and the composite component analysis method to derive the flexural and axial compression rigidity of the profile during the elastic stress stage. The flexural capacity for this type of PGWC profile is 3.3-fold the sum of the flexural capacities of the wood core and the glass-fiber-reinforced polymer (GFRP) shell. The equivalent flexural rigidity is 1.5-fold the summed flexural rigidity of the wood core and GFRP shell. The maximum axial compressive bearing capacity for this type of PGWC profile can reach 1.79-fold the sum of those of the wood core and GFRP shell, and its elastic flexural rigidity is 1.2-fold the sum of their rigidities. These results indicate that in PGWC profiles, GFRP and wood materials have a positive combined effect. This study produced a pultruded composite material product with excellent mechanical performance for application in structures that require a large bearing capacity.

## Introduction

Pultrusion technology is a continuous manufacturing process for the production of constant cross-sectional composite profiles. In addition to its high mass-weight ratio and the corrosion resistance of the composite material itself, pultruded fiber-reinforced polymer or fiber-reinforced plastic (FRP) profiles also offer good longitudinal tensile performance, arbitrary designability in section shapes, stable product quality, good aesthetic appearance, and high productivity, and such profiles have been increasingly applied in structural engineering [1–4]. Generally speaking, after unidirectional (UD) roving and continuous filament mat (CFM) layers are impregnated with a polyester resin, they are fed into a die and cured at a high temperature to form a pultruded FRP profile [5, 6]. Although the pultrusion process is, in principle, a simple one, pultrusion faces certain crucial challenges in practice, such as residual stresses in the product that may induce damage or premature cracking and delamination [7–9]. Scholars have conducted many studies on the pultrusion process, as well as the basic mechanical performance of pultruded FRP profiles, their corrosion resistance, and their creep properties [10–15]. Furthermore, the durability of a pultruded profile places key constraints on its engineering applications; therefore, this property is of widespread concern. Depending on the application, the environmental factors involved include primarily alkali aqueous environments, ultraviolet radiation, humidity, and freezing conditions [16–18]. Moreover, the use of different types of resin can have a significant impact on the durability of a pultruded profile. Carra and Carvelli [18] conducted 6-month accelerated artificial aging tests and 12-month natural aging tests of three different types of pultruded glass-fiber-reinforced polymer (GFRP) profiles with isophthalic polyester, orthophthalic polyester and vinylester as their base materials. The results indicated that the pultruded GFRP profiles with the three different types of base materials all followed the same attenuation law and that in most cases, the pultruded GFRP profile with isophthalic polyester as the base material exhibited the smallest attenuation. In addition, regarding the basic mechanical performance of pultruded profiles, the available research results show that under an external load, the plate of a pultruded FRP profile is likely to undergo local buckling failure and cleavage failure. Di Tommaso and Russo conducted an axial test of an I-shaped cross-sectional pultruded profile and studied the local buckling and longitudinal cracking of the plate [19]. Hashem et al. studied the plate-buckling phenomenon for a short pultruded FRP profile column with a universal cross section under axial compression [20]. Wu et al. conducted a bending test of a pultruded GFRP profile and studied the local buckling and longitudinal cracking of the plate. These two failure modes severely affect the overall strength of an FRP profile and lower the service efficiency of FRP materials [21].

To improve the mechanical performance of a pultruded FRP profile, Zi et al. filled a prefabricated pultruded FRP profile possessing multiple rectangular openings with polyurethane foam, thereby obtaining a sandwich composite component, and conducted a longitudinal bending and transverse bending experiment [22, 23]. The transverse bending test showed that the flexural rigidity and bearing capacity of the component with the foam filling were increased by a factor of at least 2 compared with the component without foam filling. The longitudinal bending test showed that the two types of specimens exhibited different failure modes. Buckling failure occurred at the central web of the specimen without foam filling, whereas no such buckling failure was observed for the specimen with foam filling. This difference in behavior can be primarily attributed to the support provided by the foam, similar to a Winkler foundation supporting a beam. Compared with the specimen without foam, the bearing capacity of the specimen with foam was improved by nearly 23%. Zi et al. also noted that the flexural rigidity did not change significantly, primarily because of the small elastic modulus of the foam [22].

In addition, Thomas et al. [24, 25] and Wang et al. [26] conducted bending tests and axial compression tests, respectively, on light wood composite components to determine their flexural and compressive bearing capacity and rigidity. Wang et al. also noted that increasing the density of the wood core could significantly improve the compression rigidity of a column [26]. These studies inspired us to investigate whether light wood materials with good mechanical performance could be adopted as inner filling materials for pultruded FRP components to further improve the overall mechanical performance of such components. Using prefabricated core material and overall pultrusion, Shi et al. successfully produced a new pultruded FRP profile with pultruded GFRP as the shell and a paulownia wood core as the filling and applied double cantilever beam tests to evaluate the strain energy release rates of the GFRP and the light wood interface [27]. They found that the strain energy release rate at the interface of the specimen produced via pultrusion was similar to that for a similar component produced using the vacuum infusion process. However, they did not study the bending and axial stress performances of this new pultruded GFRP profile or the effect of introducing light wood on its mechanical performance.

To study the basic mechanical performance of this new type of pultruded GFRP profile with a light wood core, including its bending performance and axial compression performance, three-point bending tests and axial compression tests were conducted in this study to determine the bending mechanical behavior, axial compression mechanical behavior and failure modes of such profiles. The Timoshenko beam theory and the composite components analysis method were used to perform the mechanical calculations for the bending and axial compression tests of the specimens, and the flexural rigidity and axial compression rigidity during the elastic stress stage were thus determined. This study can provide a method of calculating the elastic bending rigidity and axial rigidity for this type of component and can serve as a reference for further research on pultruded composite sandwich profiles.

## Pultruded GFRP-Wood Composite Profile

### Specimen design

The raw materials used in this new type of pultruded GFRP-wood composite (PGWC) profile included alkali-free glass fiber filaments, chopped strand mat, and unsaturated phthalate resin. The resin was mixed in advance with a phosphoric acid ester release agent, the content of which was approximately 1.5% of the resin mass, and the density of the paulownia wood core was approximately 270±20 kg/m^3^.

**Fig 1:**
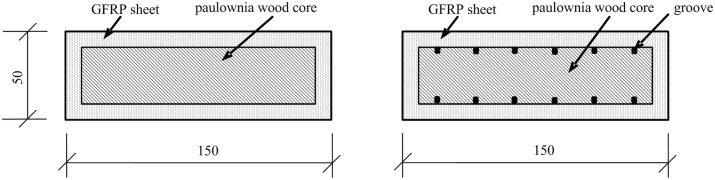
Cross sections of the PGWC profiles (mm). (a) Non-grooved PGWC. (b) Grooved PGWC.

The cross-sectional composition and dimensions of the PGWC profile are shown in Fig 1. The shell thickness of the GFRP was 5 mm, and the central main body contained one-way glass fiber filaments perpendicular to the cross section, which were the primary mechanical component. A layer of glass fiber chopped strand mat was placed on each side to improve the transverse mechanical properties of the composite material and to increase the interfacial bonding strength. The core cross section was a 140 × 40 mm rectangle. The corners were prepared using a 3-mm-radius chamfering treatment. The surface of the paulownia wood core was treated using two methods. One method was to groove the surface orthogonally in the vertical and horizontal directions. The grooves were 2.5 mm wide and 3 mm deep, with a spacing of 25 mm. The other method was to leave the surface without grooves. The PGWC profiles with these two different cross sections are denoted by G (grooved) and NG (non-grooved).

### Pultrusion

The pultrusion process for the composite sandwich component was similar to that for an FRP profile. The basic procedure was as follows. Through the traction of the power system, the fiber and core materials were constantly fed into the first terminal on the production line. First, the fiber and core materials successively traveled through the impregnation vessel, and the guiding mold and heating die were then cut to the required lengths at the end of the production line. The curing of the resin was completed primarily in the heating die.

The universal mold for manufacturing pultruded FRP profiles required only an external mold, with a cross-sectional inwall size of 150 × 50 mm and a length of 900 mm. This external mold possessed a preheating zone, a gel zone and a curing zone, with heating temperatures of 140, 160 and 145°C, respectively. Through repeated experiments, the optimal setting for the pultrusion speed was determined to be 10 cm/min.

**Fig 2:**
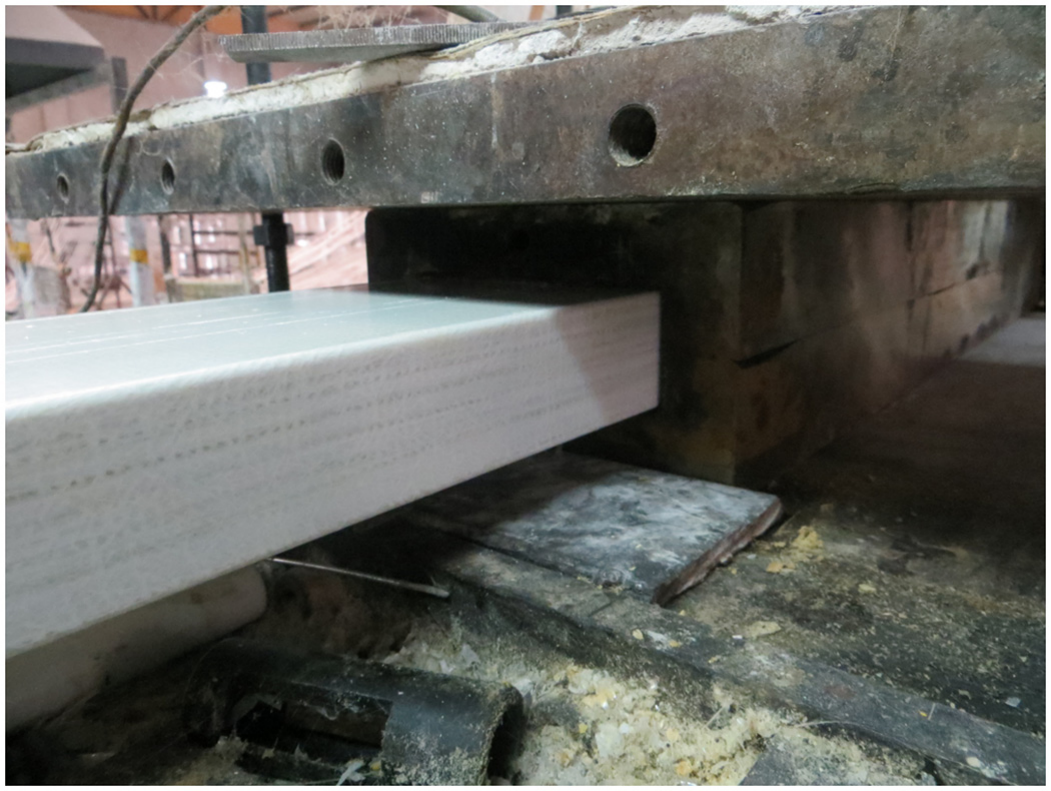
Sandwich plate exiting the die.

Fig 2 shows the sandwich plate exiting the die in the pultrusion experiment.

## Research Plan

### Description of specimens

Specimens of two lengths were produced for the experiment (Table 1). Three-point bending tests and axial compression tests were conducted to determine the basic bending and axial mechanical performances, respectively, of the PGWC profile. The specimens required for the experiment were cut from the produced PGWC profile. The specimen length for the three-point bending tests was 850 mm, and that for the axial compression tests was 400 mm. In addition, two other components were investigated for comparison: a pure wood component W, with material and dimensions identical to those of the core material in the NG series, and a pultruded GFRP rectangular pipe P, with material and dimensions identical to those of the GFRP shell in the NG series. A letter B in a specimen label indicates that the specimen was used for a three-point bending test, whereas a letter C in a specimen label indicates that the specimen was used for an axial compression test. Three identical specimens of each type of experimental component were tested.

**Table 1:** Specimen parameters.

Type	Dimensions (mm)				Number of specimens (n)
	Length	Width	Height	Thickness of GFRP	
NG-B	850	150	50	5	3
G-B	850	150	50	5	3
W-B	850	140	40	0	3
P-B	850	150	50	5	3
NG-C	400	150	50	5	3
W-C	400	140	40	0	3
P-C	400	150	50	5	3

### Material properties

All of the mentioned specimens consisted of two base materials, namely, GFRP and wood.

In accordance with the testing methods [26] specified by ASTM D3039/D3039M-08 [28], ASTM D695-10 [29] and ASTM D4255/D4255-01(2007) [30], tensile tests, compression tests and in-plane shear tests were conducted on GFRP specimens to determine the tensile strength and modulus, the compression strength and modulus, and the shear strength and modulus, respectively. Five valid specimens were subjected to each type of test, and the test results are shown in Table 2.

**Table 2:** Test results for GFRP and wood properties.

	Longitudinal tensile performance		Longitudinal compressive performance		Transverse shear performance	
	Strength (MPa)	Modulus (GPa)	Strength (MPa)	Modulus (GPa)	Strength (MPa)	Modulus (GPa)
GFRP	389.4 (5.4%)*	28.51 (7.2%)	204.0 (6.9%)	28.32 (7.3%)	8.45 (9.6%)	3.12 (6.2%)
Wood	49.2 (7.0%)	--	21.5 (9.3%)	4.82 (7.3%)	4.97 (7.7%)	0.46 (5.6%)

*: The coefficients of variation are shown in parentheses.

The timber used in this experiment was dried paulownia wood, with an average density of 269.3 kg/m^3^ (250–300 kg/m^3^) and a coefficient of variation of 4.5%. This type of timber is an anisotropic material, and there is a large difference between the short grain performance and the transverse grain performance. The wood cores used in this experiment were in the short grain placement. Therefore, following the test methods specified in ASTM C297/C297M-04(2010) [31], ASTM C365/C365M-11a [32] and ASTM C273/C273M-11 [33], the wood was tested for its tensile, compression and shear performance in the short grain direction. Five valid specimens were tested to measure each parameter, and the test results are shown in Table 2.

### Test setup and instrumentation

#### Bending test

**Fig 3:**
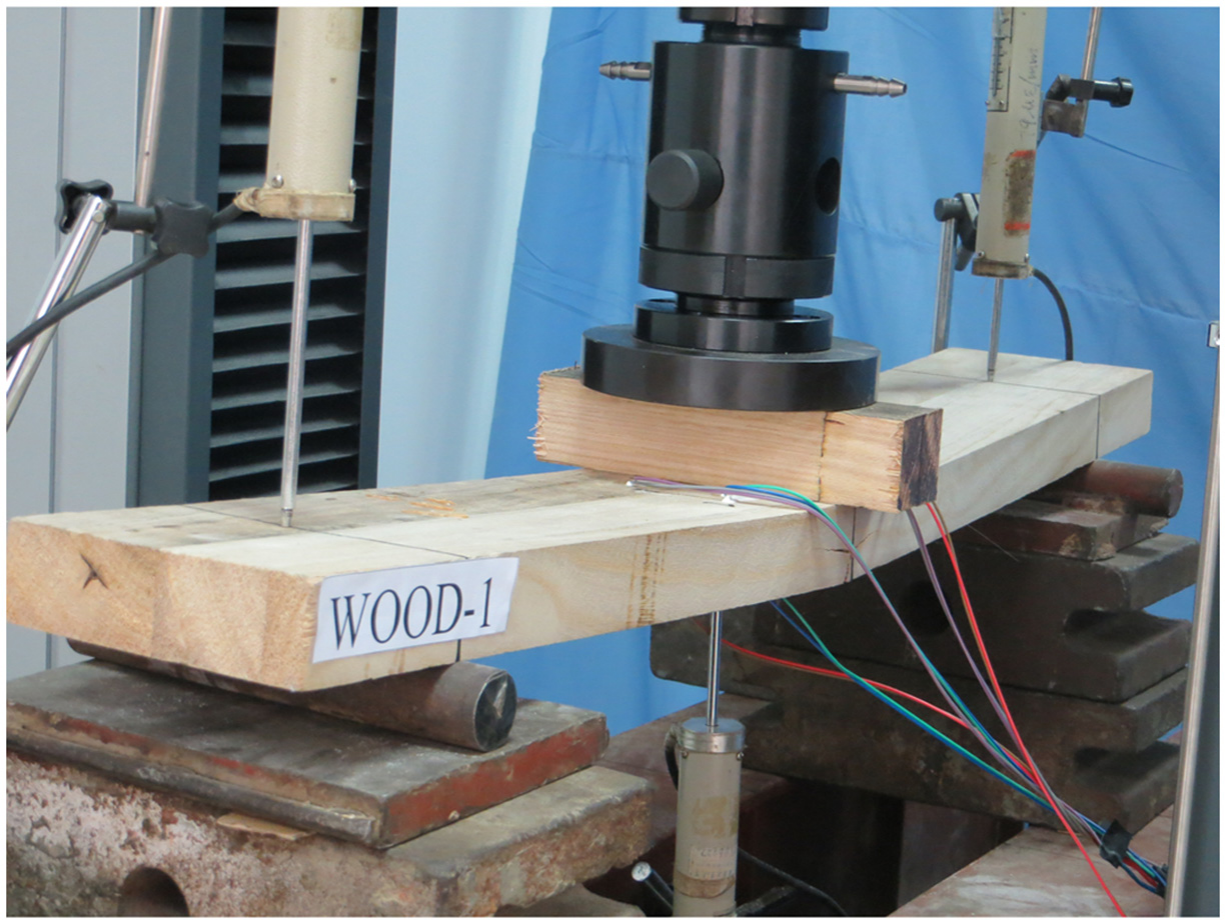
Test setup and measurement (size: mm).

Three-point bending tests were conducted to study the bending performance of the PGWC profiles [22] (Fig 3). For each specimen, a fixed hinge bearing was placed on one end and a sliding bearing was placed on the other. The distance from the end of the specimen to the bearing was 75 mm, and the net span of the specimen was 700 mm. To prevent local failure of the specimen caused by a high stress concentration near the loading point during the loading process, a fir cushion block with dimensions of 50 × 50 × 200 mm was placed between the loading point and loading head.

Three displacement transducers were used to measure the displacements near the two bearings and the midspan longitudinal displacement. The strain was measured using a strain gauge model with a sensitivity coefficient of 2.05 and an initial resistance of 120 Ω. Two strain gauges were placed at the midspan on the lower surface of the beam, and two strain gauges were placed on both sides of the fir cushion block on the top surface of the beam.

#### Axial compression test

**Fig 4:**
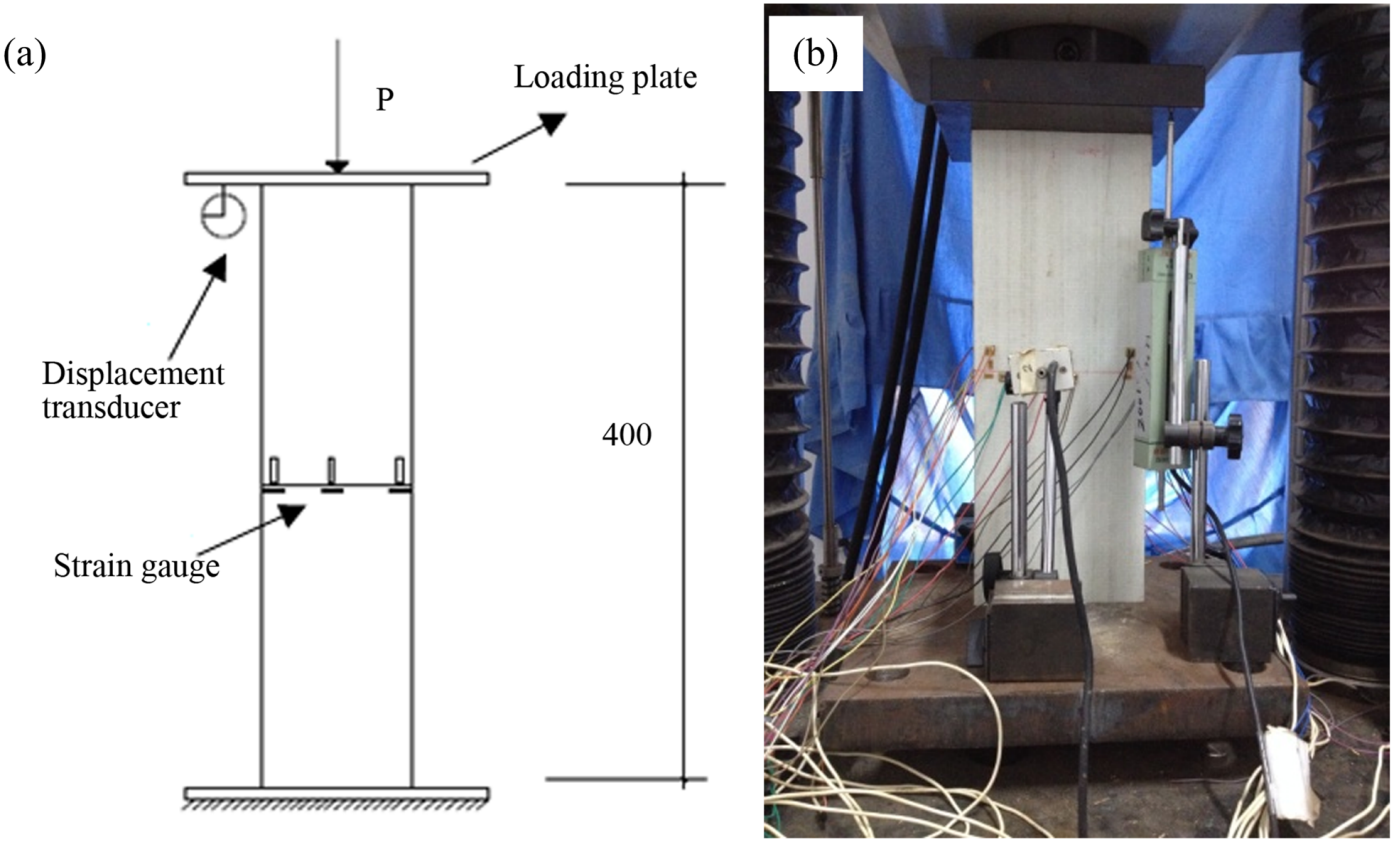
Loading and measurement setup. (a) Test setup. (b) Strain gauge on web.

A 600-kN hydraulic universal testing machine (Shenzen Sun Co., Ltd., Shenzhen, China) was used for testing (Fig 4). Before each test, the specimen was carefully centered. A steel plate of approximately 4 cm in thickness was placed on both the upper and lower sides of the specimen.

Three displacement transducers were positioned, two of which were placed symmetrically on the front and back sheets to measure the transverse displacement. The third transducer was placed vertically to measure the axial displacement. Three sets of strain gauges were placed in the middle of the specimen along the long side. One set of strain gauges was placed along the short side. Each set of strain gauges consisted of one longitudinal strain gauge and one toroidal strain gauge.

### Mechanical analysis

#### Analysis of the flexural performance

**Fig 5:**
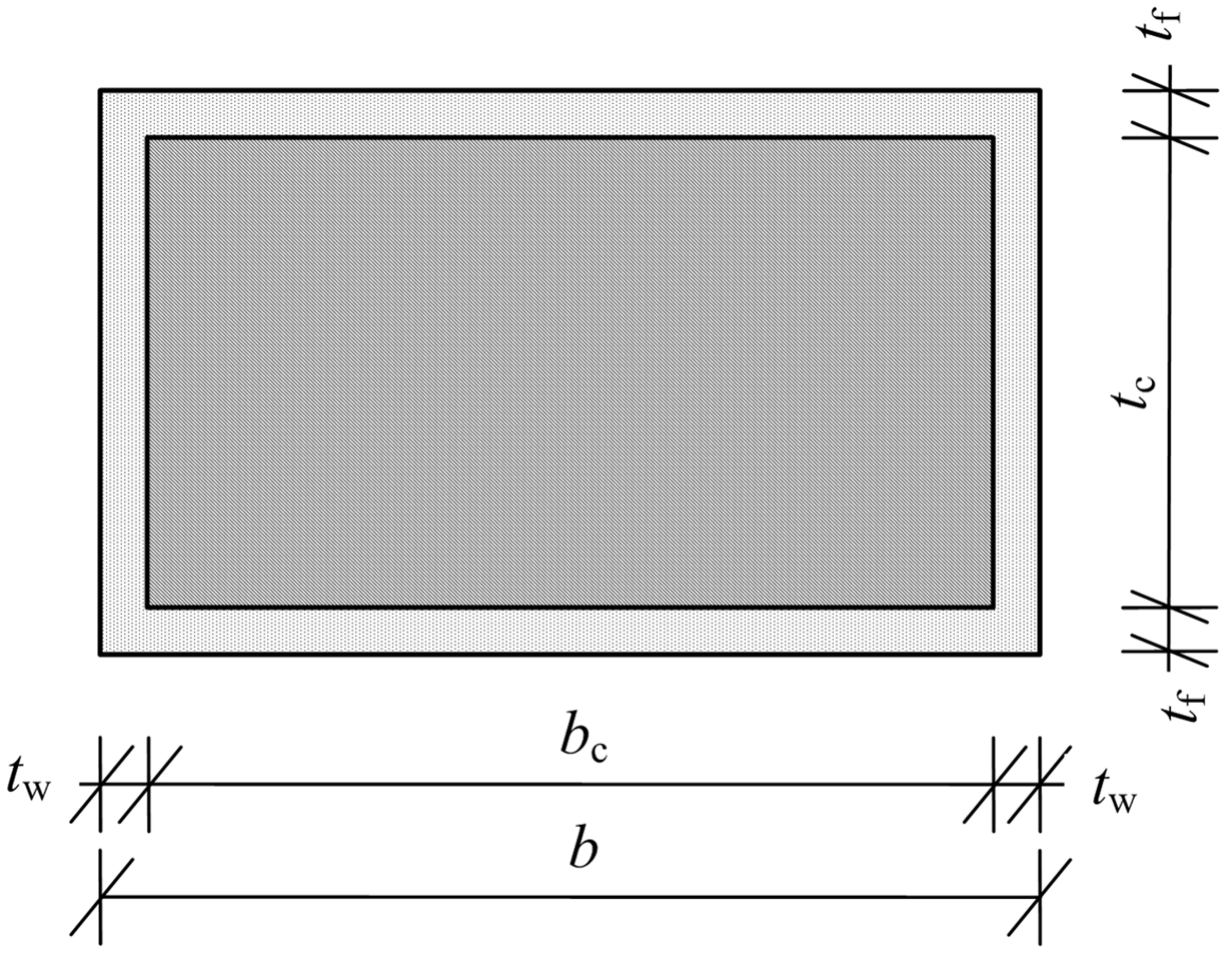
Schematic diagram of the cross-sectional geometric parameters.

The Timoshenko beam theory typically offers good solving precision for the flexural loading of a composite sandwich component [34]. Therefore, this theory was used to determine the deflection of the PGWC profile in this experiment. Using this theory, the midspan deflection *δ* can be calculated using the following expression:

$$\delta =\frac{Pl^{3}}{48{\left(\text{EI}\right)}_{s}}+\frac{Pl}{4(\text{AG)}}$$ (1)

where (EI)_s_ and AG are the equivalent flexural rigidity and equivalent shear rigidity, respectively, and can be calculated using the following expressions:

$${\left(EI\right)}_{\text{s}}=E_{\text{f}}\frac{bt_{\text{f}}^{3}}{6}+E_{\text{f}}\frac{bt_{\text{f}}^{}{\left(t_{\text{c}}^{}+t_{\text{f}}^{}\right)}^{2}}{2}+E_{\text{w}}\frac{t_{\text{w}}^{}t_{\text{c}}^{\text{3}}}{6}+E_{\text{c}}\frac{b_{\text{c}}t_{\text{c}}^{3}}{12}$$ (2)

$$AG=2t_{\text{w}}^{\prime }(t_{\text{c}}+t_{\text{f}})G_{\text{w}}$$ (3)

where *t*_f_, *t*_w_ and *t*_c_ are the thicknesses of the sheet, web and core, respectively; *b* is the width of the cross section; *b*_c_ and *t*_c_ are the width and thickness, respectively, of the core (Fig 5); *E*_f_, *E*_w_ and *E*_c_ are the elastic moduli of the sheet, web and core, respectively; the parameter *t*_w_' is the width of the equivalent web; and *G*_w_ and *G*_c_ are the shear moduli of the web and core, respectively. Thus, the following expression can be obtained:

$${t^{\prime }}_{\text{w}}=t_{\text{w}}+G_{\text{c}}b_{\text{c}}/2G_{\text{w}}$$ (4)

#### Analysis of the axial compression performance

The following basic assumptions were adopted in the mechanical analysis:

1. When a composite column is under compression, the wood core and GFRP sheet are jointly deformed, i.e., they have the same compressive strain.
2. When a composite column is under compression, the contribution of the interaction between the wood core and the GFRP sheet to the bearing capacity and rigidity of the column can be denoted by the combination coefficient *φ*.

Based on these assumptions, the axial force *N* sustained by the composite column can be expressed as follows, according to the literature [35]:

$$\text{N=(1}+\varphi \text{)}({\text{N}}_{\text{w}}{\text{+N}}_{\text{f}})$$ (5)

where *N*_*w*_ and *N*_*f*_ are the axial forces sustained by the wood core and the GFRP, respectively, and can be expressed as follows:

$${\text{N}}_{w}=E_{w}\varepsilon_{w}{\text{A}}_{w},{\text{N}}_{f}=E_{f}\varepsilon_{f}{\text{A}}_{f}$$ (6)

where *E*_*w*_, *ε*_*w*_ and *A*_*w*_ are the elastic modulus, compressive strain and cross-sectional area of the wood core, respectively, and σ_*f*_, *ε*_*f*_ and *A*_*f*_ are the elastic modulus, compressive strain and cross-sectional area of the GFRP sheet, respectively.

The longitudinal compressive strain of the column is denoted by *ε*; therefore, according to the first basic assumption, we know that the following expressions are true:

$$\varepsilon_{w}=\varepsilon_{\text{f}}=\varepsilon \text{ and }\varepsilon =\frac{\Delta }{L_{0}}$$ (7)

Expressions (6) and (7) can be substituted into Expression (5) to derive the following:

$$\text{N=(1}+\varphi \text{)}({\text{E}}_{w}{\text{A}}_{\text{w}}+{\text{E}}_{\text{f}}{\text{A}}_{\text{f}})\frac{\Delta }{{\text{L}}_{0}}$$ (8)

Using Eq (8), the compression displacement Δ can be solved for any arbitrary axial force *N*.

## Results and Discussion of the Bending Tests

### Test outcomes

#### W-B Specimens

**Fig 6:**
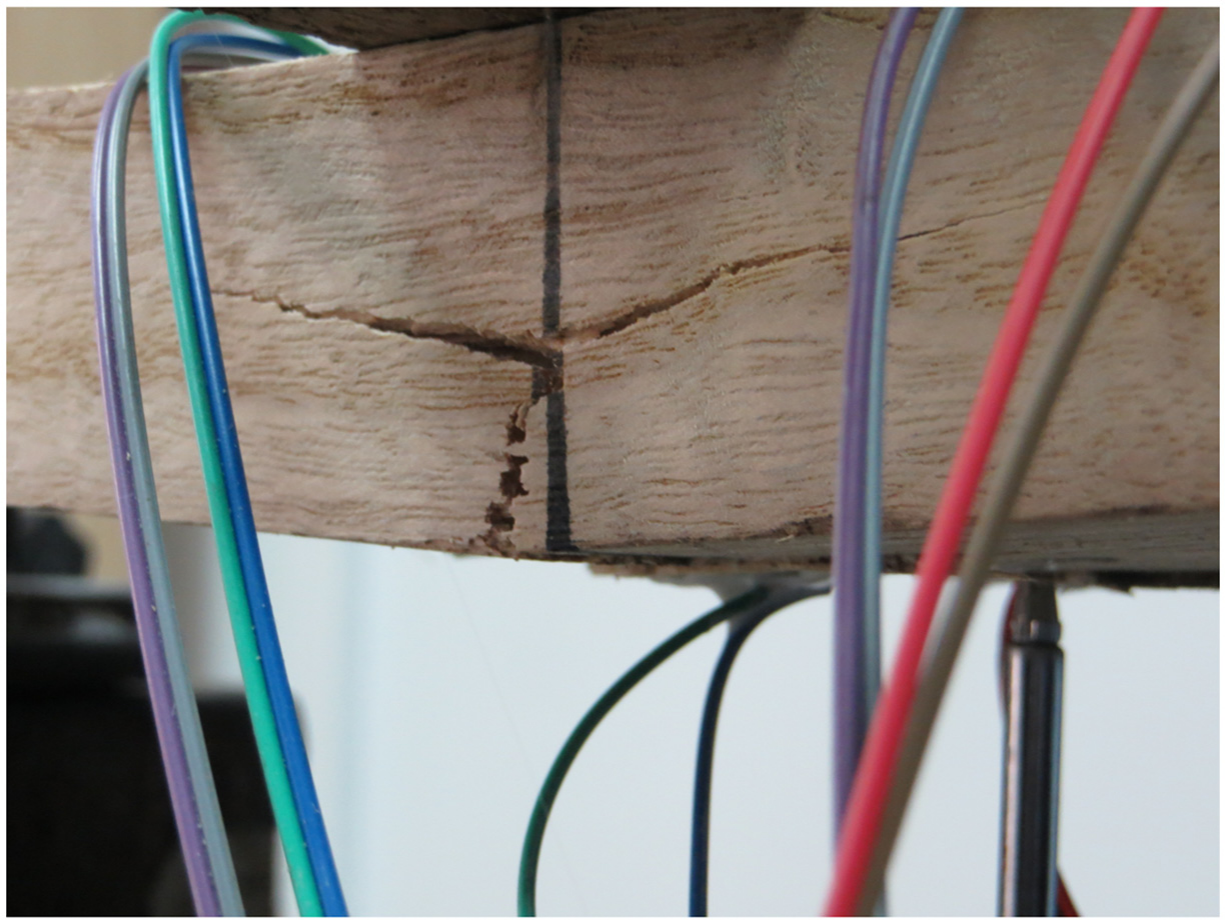
Failure mode of paulownia.

Upon loading, the specimen deflection increased with increasing load. When the load reached 2.7 kN, a sudden, loud cracking sound was heard, and the load decreased to 2.4 kN (Fig 6). At this time, the wood in the tensile region of the underside of the beam cracked, forming irregular transverse cracks, and once the cracks had expanded to approximately the half-height of the beam, the cracks extended to the oblique upward side on both sides along the wood grain; the deflection was approximately 11.78 mm, approximately 1/59 of the beam span. With continued loading, the load initially decreased and then increased slightly but rapidly. The cracks expanded along the wood grain to the downward side of the loading point. When the deflection was 58.7 mm (approximately 1/12 of the span), the loading was terminated.

#### P-B Specimens

**Fig 7:**
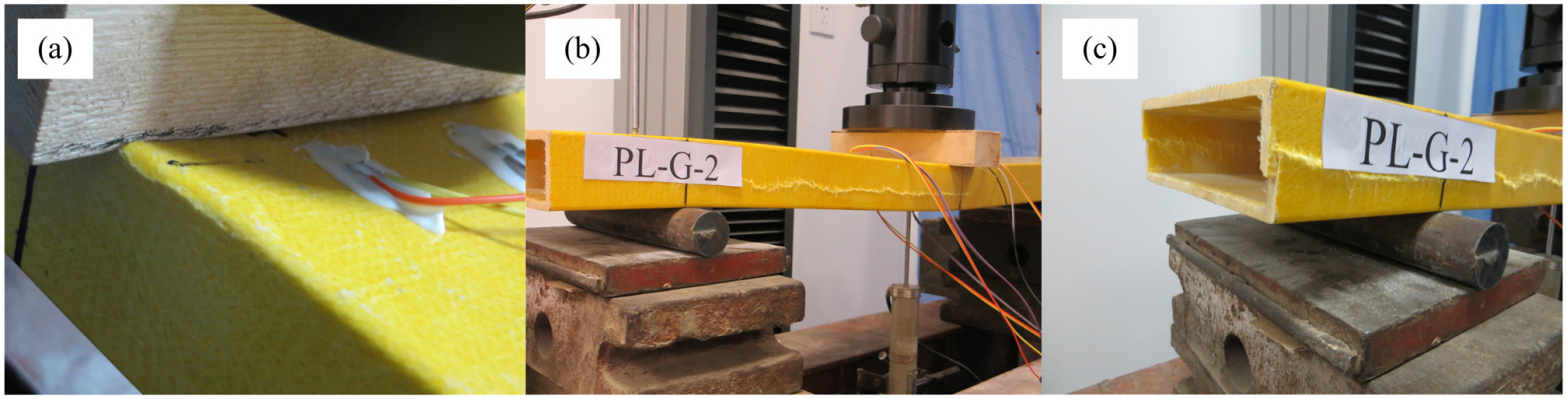
Failure mode of P-B specimens. (a) Fiber cleavage at the corner. (b) Fiber cleavage in the web. (c) Split to the end of the beam.

Upon loading of a P-B specimen, the GFRP sheet beneath the loading point bowed downward and separated from the loading block, causing the loading block to exert the load directly onto the webs on both sides, and the concavity increased with increasing load. This was because there was no supporting core within the component, which was a typical thin-walled hollow component. Under the action of the transverse load, a transverse bending moment was applied to the upper and lower sheets, and because the transverse flexural capacity of the FRP sheets was quite weak, obvious concave deformation of the upper sheet occurred. When the load reached 11.5 kN, the pipe suddenly produced a loud cracking sound, and the load abruptly decreased. The initial failure occurred at the junction where the web and the upper sheet intersected. Because the transverse stress at this location was rather large, transverse cracks first formed in the matrix (Fig 7(a)). With continued loading, constant cracking sounds were heard from the GFRP, and the cleavages expanded to both ends of the beam. The deflection increased significantly, the load declined continuously, and the webs on both sides beneath the loading point bulged slightly outward. When the beam was loaded to a deflection of 7.5 mm, the GFRP produced a clear tearing sound, and horizontal cleavages appeared on the lower side on one side of the web (Fig 7(b)). With continued loading, the load remained nearly unchanged, and the deflection increased rapidly. When the deflection reached 14 mm (approximately 1/50 of the span), the horizontal cleavages of the webs on both sides split to both ends (Fig 7(c)), and at this time, the loading was terminated. This experimental result was similar to that reported by Wu et al. in the literature [21], in which case the failure occurred at the edge at the intersection between the top surface beneath the loading point and the web, and the web exhibited horizontal cracks. The primary causes of this phenomenon were that the pultruded GFRP profile was a thin-walled component and the main fibers (in addition to the FRP sheet, there was a layer of fiber felt on both the external and internal surfaces) that sustained the stress were distributed along the lengthwise direction of the beam. Therefore, the transverse mechanical performance was relatively weak.

#### NG-B Specimens

**Fig 8:**
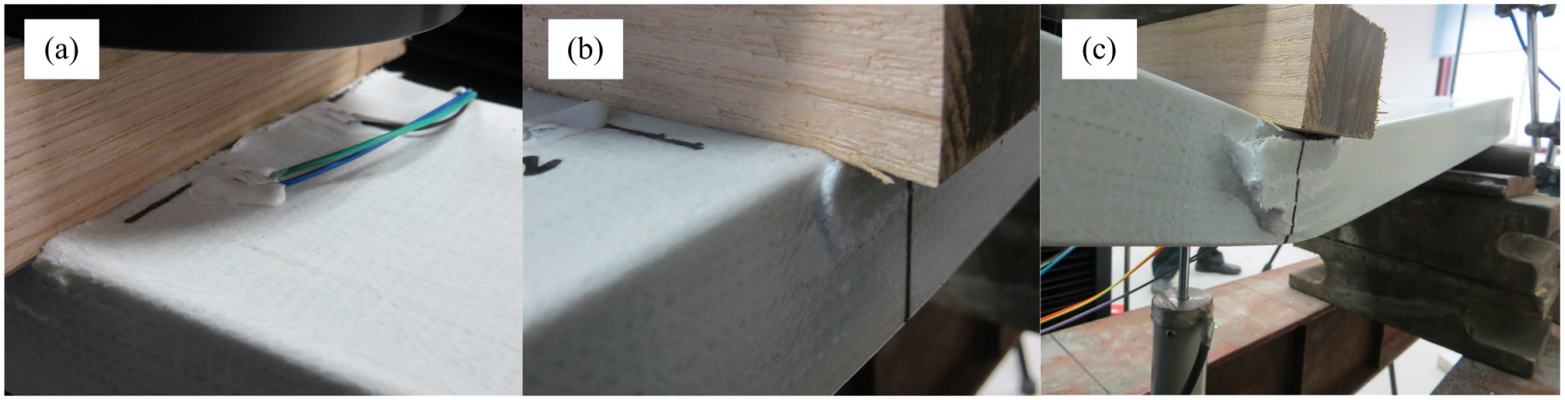
Failure mode of NG-B specimens. (a) Surface rupture. (b) Web buckling. (c) Web damage.

The deflection of the NG-B specimens increased with increasing load. When the load reached 41 kN, the specimen produced a slight snapping sound near the loading point; however, there was no visible cleavage. Therefore, both the load and deflection could continue to increase. When the load reached 47 kN, a loud rupturing sound suddenly occurred, and the load decreased to 19 kN. At this time, a transverse rupture on the top sheet of the specimen developed along the loading block boundary, and the rupture expanded to the webs on both sides (Fig 8(a)). With continued loading, the deflection increased rapidly, and the load decreased slowly. Under loading, the webs on both sides gradually buckled outward (Fig 8(b)), indicating that the GFRP sheet had already separated from the paulownia wood core. With continued loading and displacement, the webs were damaged by the shearing force and the ruptures gradually expanded to the bottom of the beam (Fig 8(c)). When the deflection reached 52 mm (approximately 1/14 of the span), the loading was terminated. At this time, the remaining load was 13 kN. Failure occurred in the side webs of the specimen in this experiment, similar to the failure observed in a composite sandwich beam in the edgewise position in a bending test performed by Manalo [36]. However, the overall failure process was different. Because the beam tested in the cited experiment had no upper or lower sheet, under the transverse load, the upper part of the web sustained enormous pressure, and finally, compression failure occurred. In contrast, the lower part of the web sustained a large tensile force, and ultimately, tensile failure occurred. In this experiment, however, GFRP sheets surrounded the specimen. The upper and lower sheets sustained high pressure and tension, respectively, thereby lowering the positive stress of the web, and the web exhibited outward buckling failure.

#### G-B Specimens

**Fig 9:**
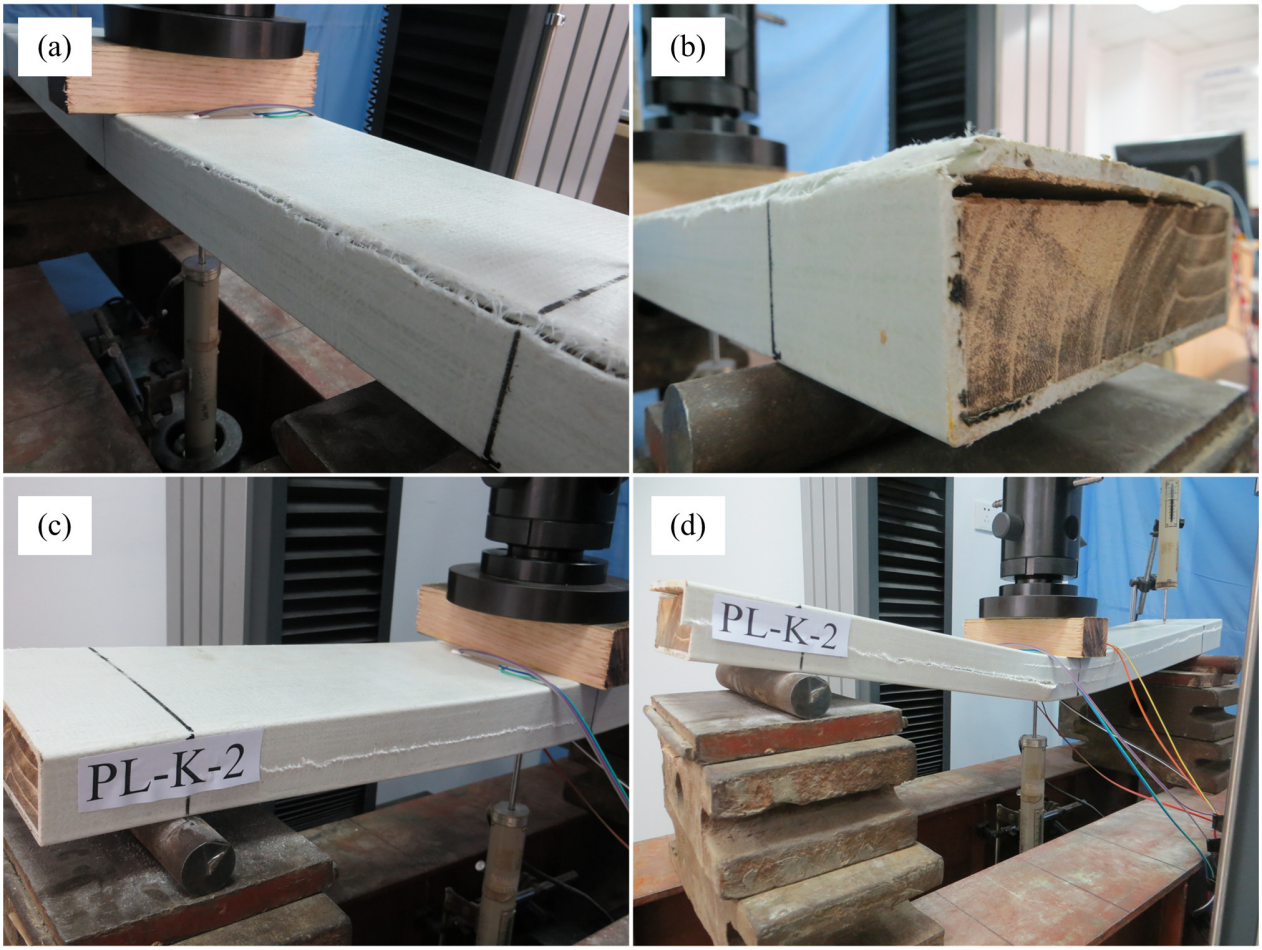
Failure mode of G-B specimens. (a) Ripping between the GFRP sheet and the web. (b) Debonding at the interface. (c) Splitting of the web. (d) Sliding between the GFRP sheets and the paulownia wood core.

During the early loading stage, the G-B and NG-B specimens demonstrated the same performance. When the load reached 34 kN, however, each G-B specimen suddenly produced a loud ripping sound from the GFRP. Horizontal ripping cracks appeared on the upper side of the GFRP web along the lengthwise direction of the beam (Fig 9(a)), and the load decreased to 22.7 kN. With continued loading, it was visible from the beam end that the upper sheet and paulownia wood core had already debonded at the interface (Fig 9(b)). However, it was possible to continue loading. When the load reached 26.2 kN, horizontal splits formed in the GFRP web on one side, stretching from underneath the loading point to the inner side of the abutment (Fig 9(c)). With continued loading, these splits expanded to the outer side of the abutment, and new horizontal splits appeared on the GFRP web and expanded toward both ends until continuous horizontal seams had formed. Sliding between the GFRP sheets and the paulownia wood core could be observed from both ends, with a length of 1 cm (Fig 9(d)). The deflection increased rapidly, and the load declined continuously. When the deflection reached 48 mm (approximately 1/15 of the span), the loading was terminated.

### Analysis of the failure modes

The four types of bending specimens exhibited different failure modes. For the wood specimens (W-B), the failure mode corresponded to tensile failure of the lower part of the wood. For the pultruded GFRP rectangular pipes (P-B), the GFRP web exhibited splitting failure along the fiber direction, primarily because of the relatively weak transverse performance due to the predominantly longitudinal fibers in the pipe. The pressure-zone GFRP sheets of the PGWC profiles in which the wood cores contained no grooves (NG-B) exhibited breaking failure under the complex stress. For the PGWC profiles with grooved wood cores (G-B), longitudinal cleavage occurred along the fiber direction at the intersection of the GFRP web and the top GFRP sheet, and the GFRP sheets and the paulownia wood core separated from each other.

A comparison of these failure modes reveals that the composite profile formed by a paulownia wood core and a hollow pultruded GFRP pipe can modify the stress distribution within the plate, changing the failure modes of the paulownia wood and the pultruded GFRP pipe.

In addition, the G-B and P-B specimens exhibited similar failure modes. Because of the grooved surface of the wood core of a G-B specimen, the resin could not completely fill the grooves on the surface of the core during the pultrusion process; as a result, cavities formed where there was insufficient resin, thereby creating an initial deficiency. When the composite sandwich plate bent as a whole, this initial deficiency expanded, leading to the separation of the paulownia wood core from the GFRP sheet and causing shearing cracks to form on the GFRP sheet. This observation reminds us that when such a component is used in a dry environment, the wood core filling may undergo drying shrinkage, thereby degrading the performance of the interface between the GFRP sheets and the wood core, ultimately leading to a failure mode similar to that of the G-B specimens and preventing the component from achieving a high flexural capacity. To avoid such a situation, when manufacturing a PGWC profile of this type, the wood core must undergo a drying treatment to reduce its water content to the stipulated limit.

### Analysis of the combined effect of the GFRP and the wood core

#### Analysis of the peak bearing capacity

**Fig 10:**
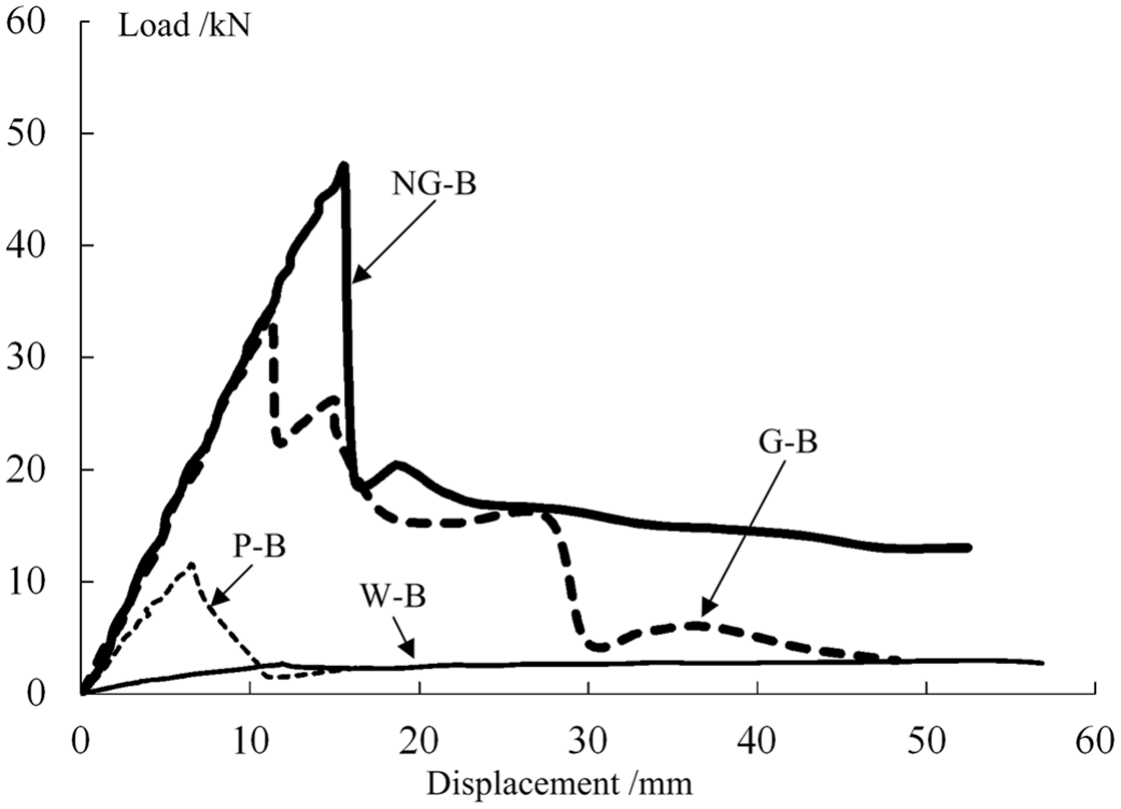
Typical load-displacement curves.

The load-deflection curves of the four types of bending specimens are shown in Fig 10. Before failure occurred, the specimens exhibited linear elasticity, and the maximum loads and the slopes in the rising segments of the curves for the NG-B and G-B specimens were significantly higher than those in the rising segments of the curves for the W-B and P-B specimens. After failure, the loads on both types of composite specimens decreased considerably, indicating that they experienced brittle failure. Then, under continued loading, the NG-B specimens exhibited good ductility, whereas after the second failure for a G-B specimen, its load decreased again, indicating that the specimen had nearly lost its bearing capacity entirely.

The load value corresponding to the apex of the load-deflection curve was regarded as the peak bearing capacity *P*_m_ for each specimen; these values are listed in Table 3. The peak bearing capacities of the PGWC profile specimens of types NG-B and G-B were respectively 47.0 and 34.0 kN, which were 17.4 and 12.6 times the peak bearing capacity of the W-B specimens, 4.1 and 3.0 times the peak bearing capacity of the P-B specimens, and 3.3 and 2.4 times the sum of the latter two values. These results indicated that the GFRP and the wood core in a PGWC profile jointly sustained the load, thereby achieving an excellent combined performance compared with that of either single material. The bearing capacity of a composite bridge deck made of a pre-pultruded GFRP core filled with foam has been found to increase by 23% compared with that of a hollow pultruded GFRP bridge deck without foam filling [23]; by comparison, this value increased by at least two-fold in this study. The bearing capacity improvement observed in this study can be primarily attributed to the better mechanical behavior of paulownia as a core compared with that of foam. Through overall pultrusion, this combination of the GFRP sheet and the inner core yielded a further improvement in bearing capacity.

**Table 3:** Experimental results.

Category	*P*_m_ (kN)	*f*_m_ (mm)	*K* = *P*_m_/*f*_m_ (kN/mm)
W-B	2.7	11.87	0.23
P-B	11.5	6.60	1.74
W+P	14.2	-	1.97
G-B	34.0	11.33	3.00
NG-B	47.0	15.60	3.01

#### Analysis of the equivalent flexural rigidity

The deflection corresponding to the peak bearing capacity *P*_m_ of each specimen is called the peak deflection *f*_m_, and the equivalent flexural rigidity *K* of the specimen is equal to *P*_m_/*f*_m_. The equivalent flexural rigidity of each type of specimen is shown in Table 3. W+P indicates the linear superposition of the values for the W-B and P-B specimens.

The equivalent flexural rigidities of the PGWC profile specimens of types NG-B and G-B were 3.07 and 3.01 kN/mm, respectively, approximately 12.8 times the flexural rigidity of the W-B specimens, 1.7 times the flexural rigidity of the P-B specimens, and 1.5 times the sum of the latter two values. These results indicate that the GFRP and the wood core in a PGWC profile jointly sustained the load, thereby achieving an excellent combined performance compared with that of either single material. The flexural rigidity of the previously cited composite bridge deck manufactured by filling a pre-pultruded GFRP core with form was nearly identical to that of the GFRP pultruded bridge deck without foam filling, as reported in the literature [23], whereas the flexural rigidity of the light wood composite component tested in this experiment represented a 70% increase compared with that of the hollow pultruded GFRP profile without the light wood core because in the component used in this experiment, the wood core not only played a role in supporting the GFRP sheet, similarly to a Winkler foundation, but also contributed to the flexural resistance of the entire beam.

In addition, the peak deflections *f*_m_ of the G-B and NG-B specimens were greater than the peak deflection of the P-B specimens. The primary reason for this finding was that during the loading process, the GFRP and the paulownia wood core jointly sustained the load. The paulownia wood core not only sustained some of the shearing force and decreased the level of stress in the GFRP web but also partially supported the web, thereby preventing the early occurrence of local buckling failure in the GFRP, which greatly improved its bearing capacity and rigidity.

Therefore, a composite beam consisting of GFRP and paulownia wood, with mechanical behavior that is markedly superior to the linear superposition of the performances of these two types of beam materials individually, can deliver an excellent combined effect.

#### Load-strain curves

**Fig 11:**
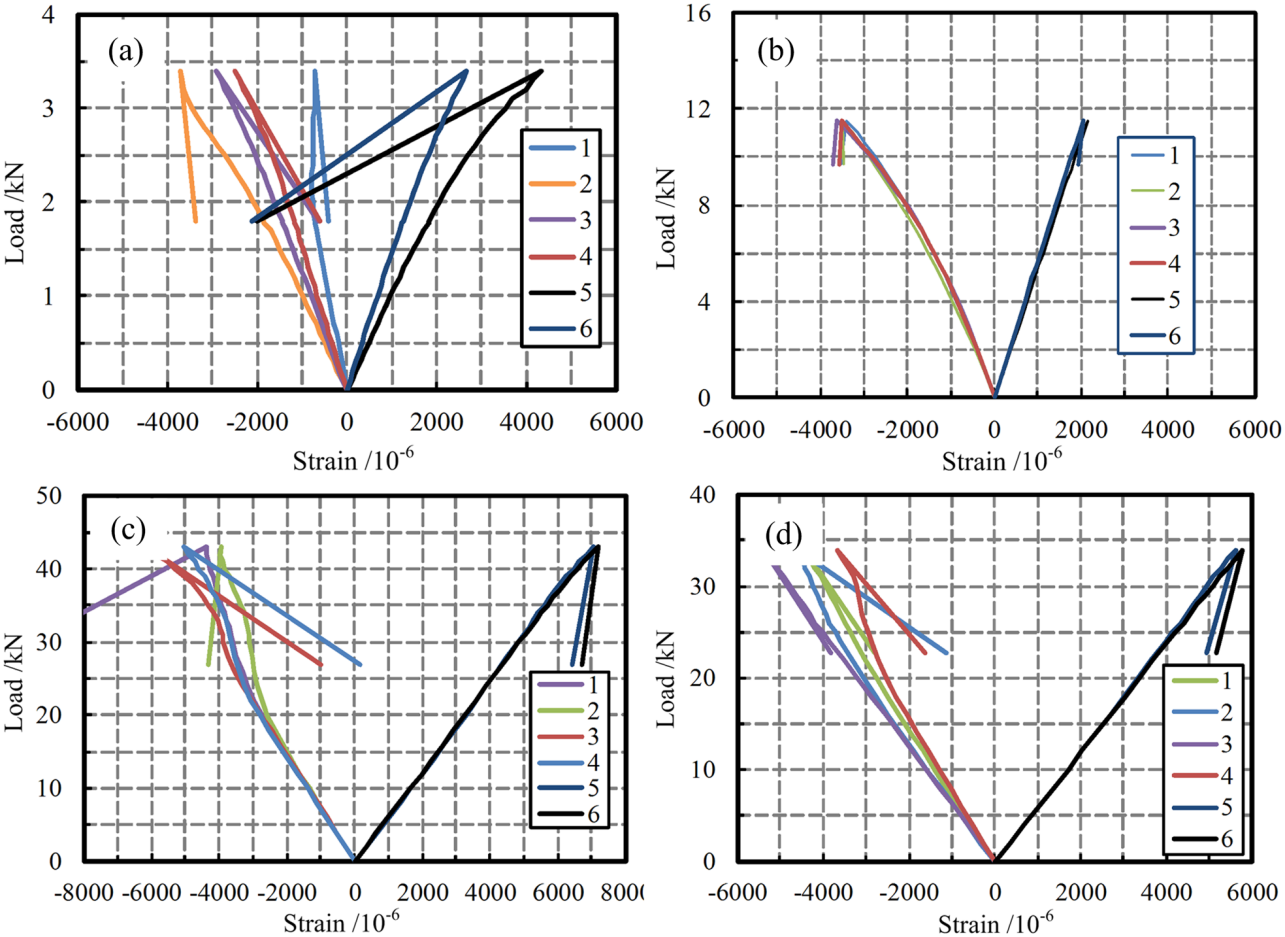
Load-strain curves. (a) W-B. (b) P-B. (c) NG-B. (d) G-B.

The load-strain curves of the lower-surface tensile zone and the upper-surface pressure zone of the midspan section are shown in Fig 11 for each of the four types of specimens. Numbers 1–6 in the figure indicate the locations of the strain-measurement points. Among these strain-measurement points, points 1–4 represent the measurement points on both sides of the upper-surface loading point, and points 5 and 6 indicate the lower-surface midspan measurement points on the specimen. The strain on the midspan section of each type of specimen varied linearly with the load, consistent with the results reported in the literature [37], indicating that the stress in this section increased evenly throughout the loading process. Fig 11 shows that the relationship between the tensile strain and the compressive strain differed for different types of specimens: The tensile and compressive strains on a W-B specimen were nearly equal. By contrast, the upper-surface compressive strain on a P-B specimen was larger than the tensile strain on the lower surface, primarily because a transverse second-order deformation occurred in the upper surface, causing the upper-surface strain to be greater than the tensile strain on the lower surface. Meanwhile, for the NG-B and G-B specimens, the upper-surface compressive strain and the lower-surface tensile strain were nearly equal, and the strain on the NG-B specimens was clearly larger than the strain on the G-B specimens. These results indicated that the GFRP material in the NG-B specimens was better utilized.

### Flexural loading

**Fig 12:**
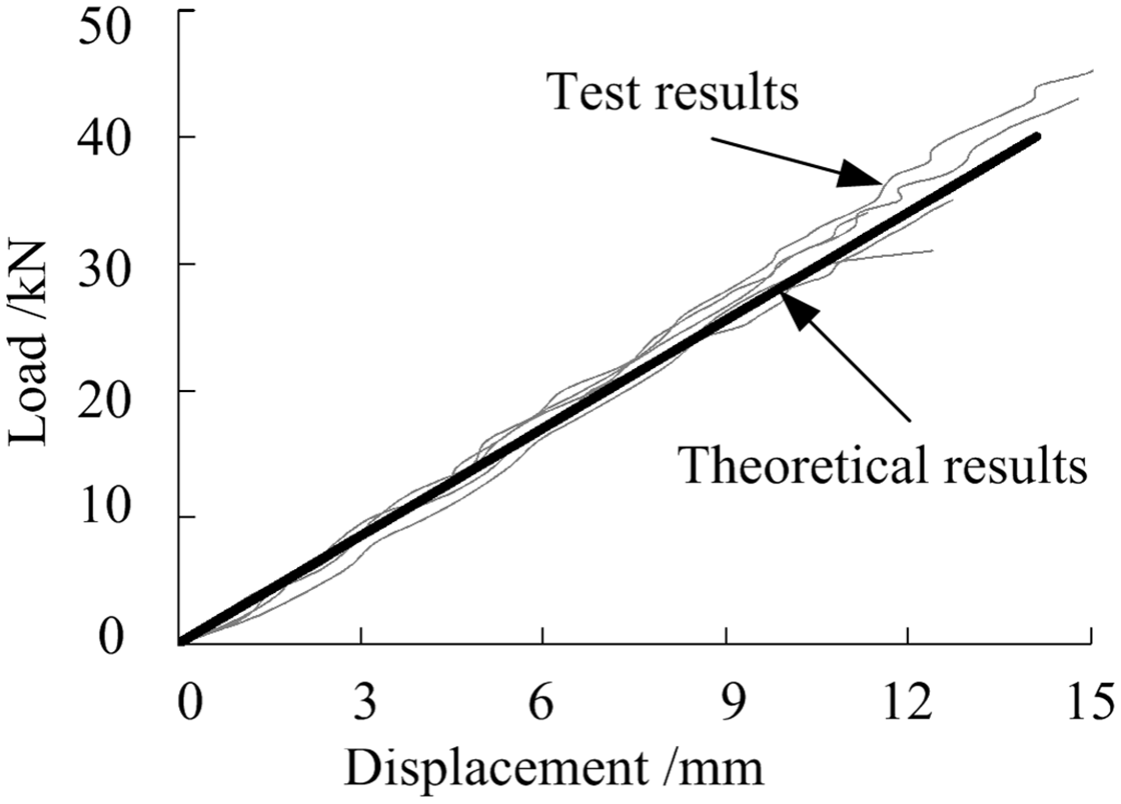
Comparison of the theoretical and experimental values for the midspan deflection of the bending specimens.

Eqs (1)–(4) were used to calculate the load and deflection of the G-B and NG-B bending specimens. The results, which are similar to the calculated results reported in the literature [36], are shown in Fig 12. The theoretical and experimental results are in good agreement, indicating that the Timoshenko beam theory can be employed for the elastic calculations for the light wood-GFRP composite beams proposed in this study.

## Analysis and Discussion of the Results of the Axial Compression Experiment

### Analysis of the failure modes

**Fig 13:**
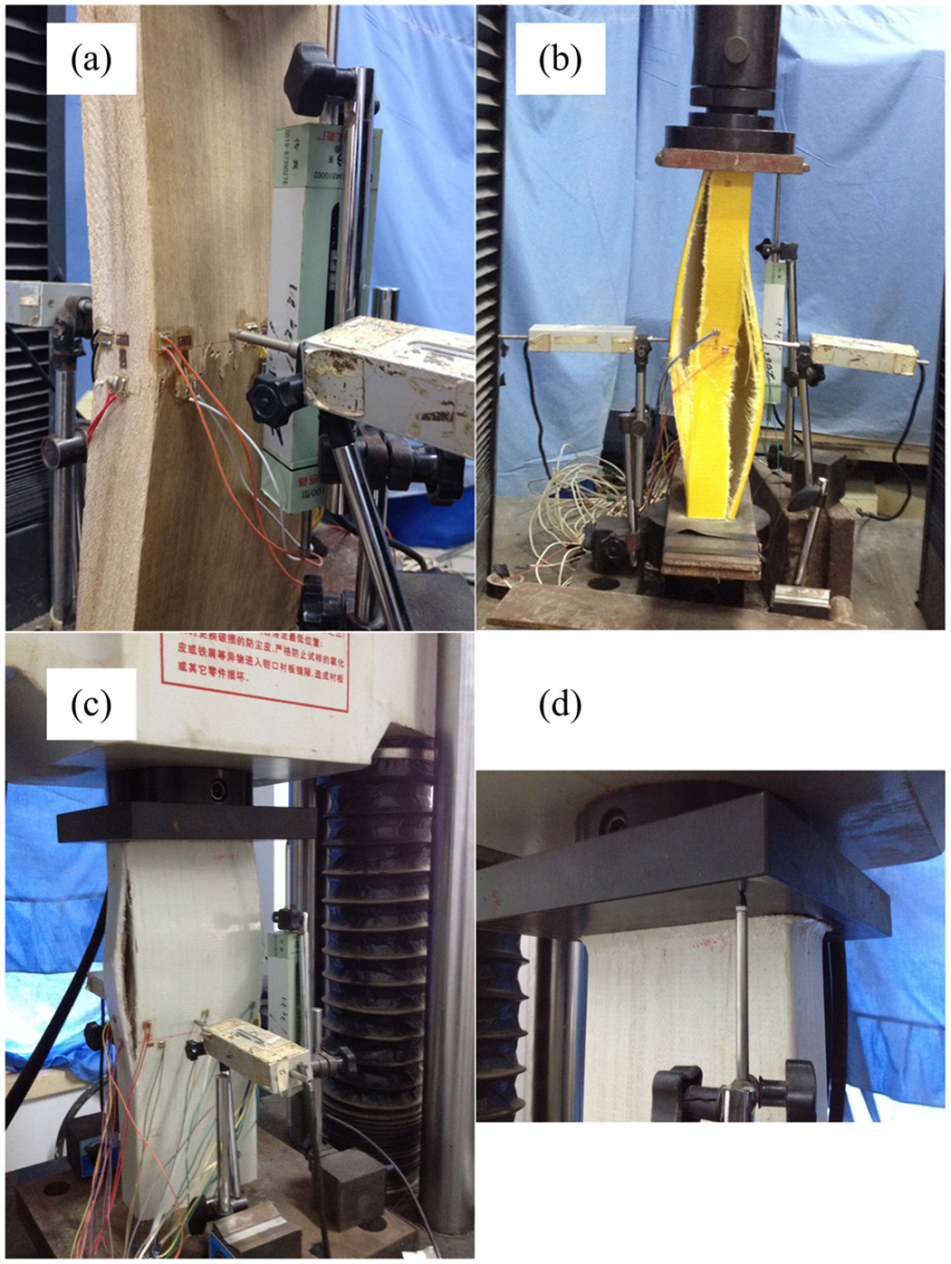
Failure modes of axial compression specimens. (a) W-C specimen. (b) P-C specimen. (c) Specimen NG-C-1. (d) Specimen NG-C-3.

The three types of specimens tested in the experiment exhibited different failure modes. The wood specimens (W-C) experienced transverse bending when the load was relatively high, and finally, the wood fiber in the central tensile zone of the specimen broke and failed (Fig 13). When a hollow pultruded GFRP profile specimen (P-C) was placed under load, the wide sheet exhibited local buckling, and finally, tearing failure occurred at the intersecting edges. This failure mode is similar to that of a “universal” cross-sectional specimen [20] and that of an I-shaped cross-sectional component [38] as described in the literature, and all of these modes represent holistic failure caused by the local buckling of the compression web. The PGWC profiles exhibited two different failure modes. When specimens NG-C-1 and NG-C-2 were placed under load, the GFRP sheet buckled outward as a whole, and tearing failure occurred at the intersecting edges. For specimen NG-C-3, crushing failure was observed in the GFRP at the loading end. The failure modes of the GFRP specimens in this experiment were nearly identical to the two types of failure modes reported in the literature for hollow sandwich columns with GFRP skins and paulownia wood cores [26]. These failure modes consist of the local buckling of the GFRP and the compression failure of the GFRP skin. The difference lies in the fact that longitudinal cracks have not previously been reported to occur after the appearance of a buckling failure in the GFRP skin [26]. However, in the present study, such cracks did appear in the specimens. The primary cause was that the fibers in the GFRP sheets of the specimens used in this experiment were distributed in a predominantly longitudinal manner, resulting in weak transverse mechanical performance and thus resulting in tearing failure due to buckling. This type of phenomenon is a common characteristic of pultruded GFRP components, which will be improved upon in the future.

A comparison of these failure modes with those of the W-C and P-C specimens reveals that the wood core of a specimen of the NG-C type had an evident constraining effect on the GFRP sheet and could prevent the GFRP sheet from caving inward; thus, it could prevent the GFRP sheet from experiencing buckling failure at an early stage. The two different failure modes of the three NG-C specimens could be primarily attributed to differences in the manufacturing of the specimens. Of these two failure modes, the occurrence of end-crushing failure corresponded to a higher bearing capacity, which inspired us to establish a rational cross-section design to avoid the outward failure mode of the GFRP sheet and thus achieve a higher bearing capacity.

### Analysis of the combined effect of the GFRP and the wood core

#### Analysis of the peak compressive bearing capacity

**Fig 14:**
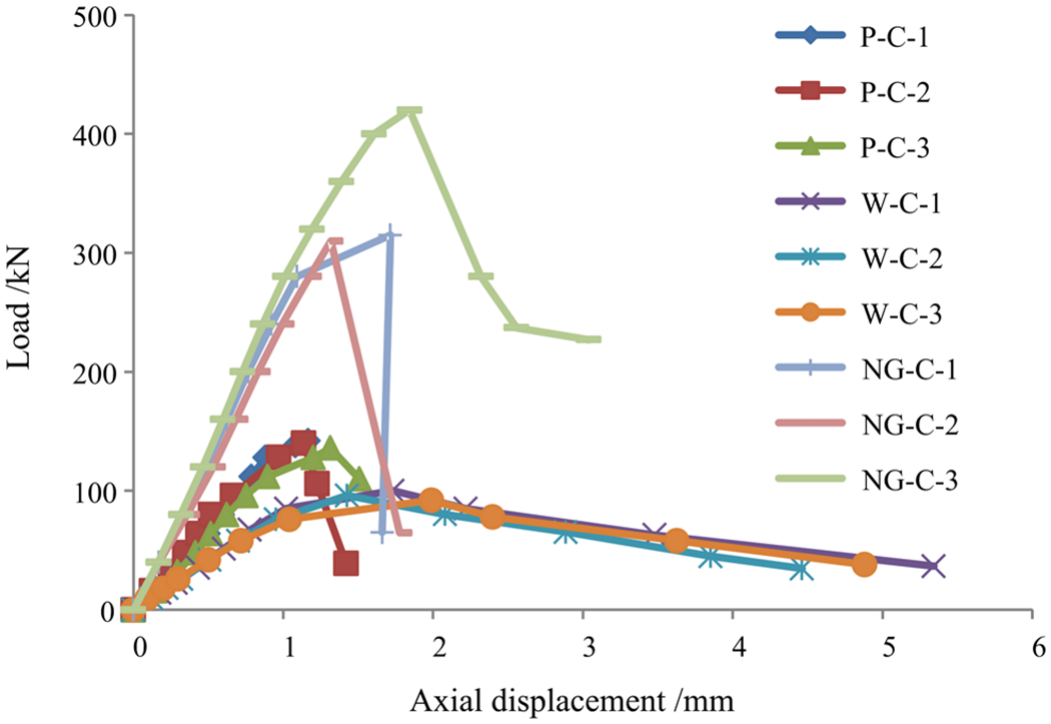
Load-displacement curves of the short columns.

The load-axial displacement curves of the three types of axial compression specimens are shown in Fig 14. The W-C specimens exhibited rather plastic characteristics before failure and suffered relatively large deformations after peak load; in addition, the decrease in the load was slow. The P-C and NG-C specimens exhibited approximately linear elastic loading characteristics; the load declined rapidly after peak load, and the specimens had low ductility.

The load value corresponding to the apex of the load-axial displacement curve for each specimen was taken to be its peak compressive capacity *P*_mc_. The average compressive capacities for the NG-C specimens that exhibited GFRP end-crushing failure and GFRP plate-buckling failure were respectively 422 and 312.5 kN, which were 4.39 and 3.25 times the average compressive capacity (96.1 kN) of the W-C specimens, 3.03 times and 2.24 times the average compressive capacity (139.3 kN) of the P-C specimens, and 1.79 times and 1.33 times the sum (235.4 kN) of the values for the W-C and P-C specimens. These results indicate that the GFRP and the wood core in a PGWC profile jointly resist axial compression loads to achieve an excellent combined performance compared with that of either single material.

#### Analysis of the elastic compressive rigidity

The loading elastic segment of each load-displacement curve was identified and used to determine the elastic compressive rigidity *K*_e_ of the specimen, as shown in Table 4. Among these values, the average value of the elastic compressive rigidity for the 3 PGWC profile specimens (NG-C) was 257.8 kN/mm, which was 3.39 times the average elastic compressive rigidity of the wood specimens (76.0 kN/mm), 1.86 times the average elastic compressive rigidity of the hollow pultruded GFRP profile specimens (138.6 kN/mm), and 1.2 times the sum of the values for the wood and hollow pultruded GFRP specimens. These results indicate that the PGWC profiles exhibited increased elastic compressive rigidity by virtue of the combined action of the GFRP and the wood core, which produced a profile with greater rigidity than the sum of its parts.

**Table 4:** Results of the axial compression experiment.

Specimen	Peak compressive capacity *P*_mc_ (kN)	Average peak compressive capacity (kN)	Elastic compressive rigidity *K*_e_ (kN/mm)	Average elastic compressive rigidity (kN/mm)
W-C-1	100.10	96.1	75.0	76.0
W-C-2	96.10		79.9	
W-C-3	92.00		73.1	
P-C-1	140.00	139.3	142.5	138.6
P-C-2	136.00		144.8	
P-C-3	142.00		128.4	
P-C & W-C	--	235.4	--	214.6
NG-C-1	310.00	312.5	260.9	257.8
N-G-C-2	315.00		234.3	
NG-C-3	422.00	422.0	278.2	

#### Load-strain curves

**Fig 15:**
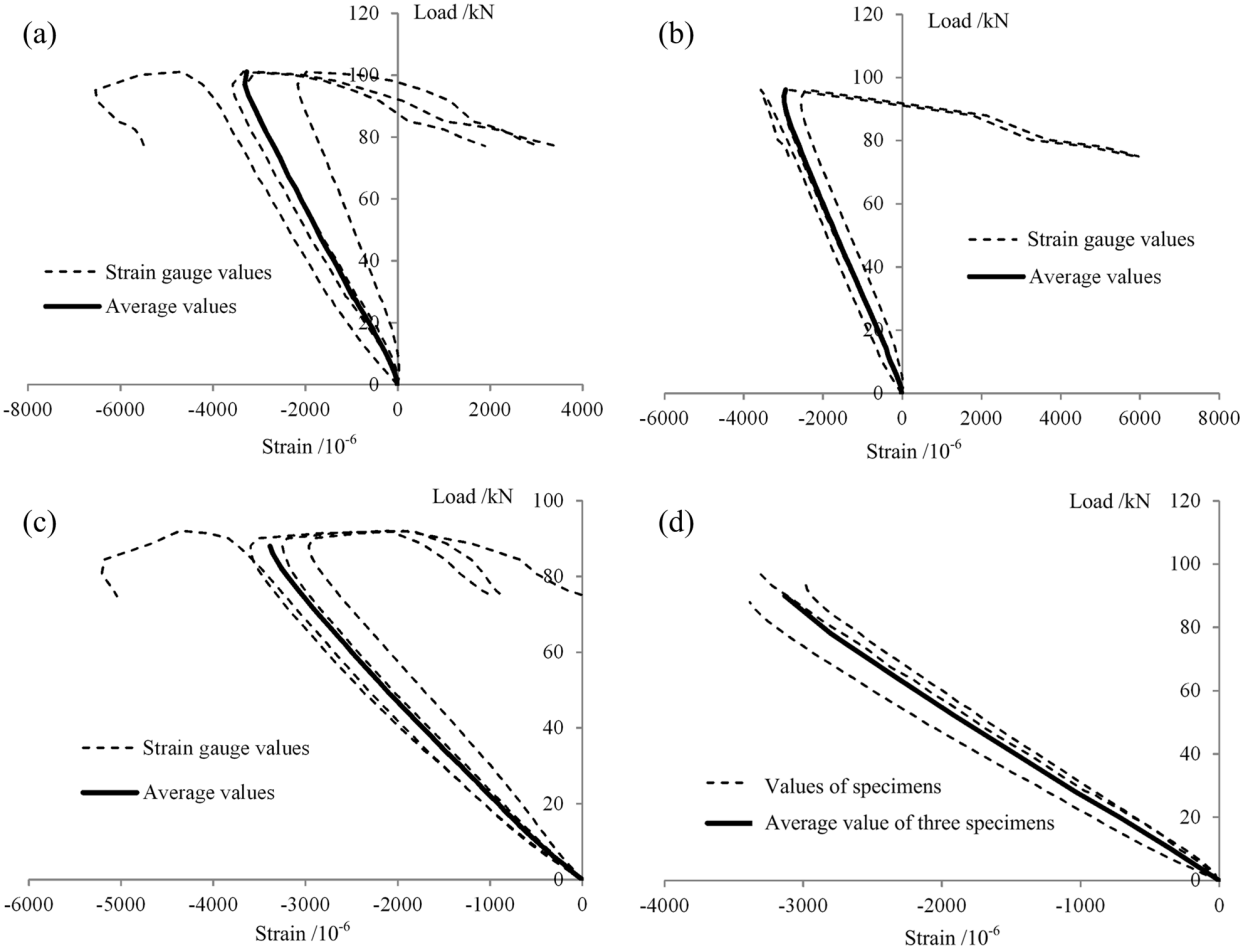
Load-axial compressive strain curves of the W-C specimens. (a) W-C-1. (b) W-C-2. (c) W-C-3. (d) Average values for all 3 W-C specimens during the elastic stage.

**Fig 16:**
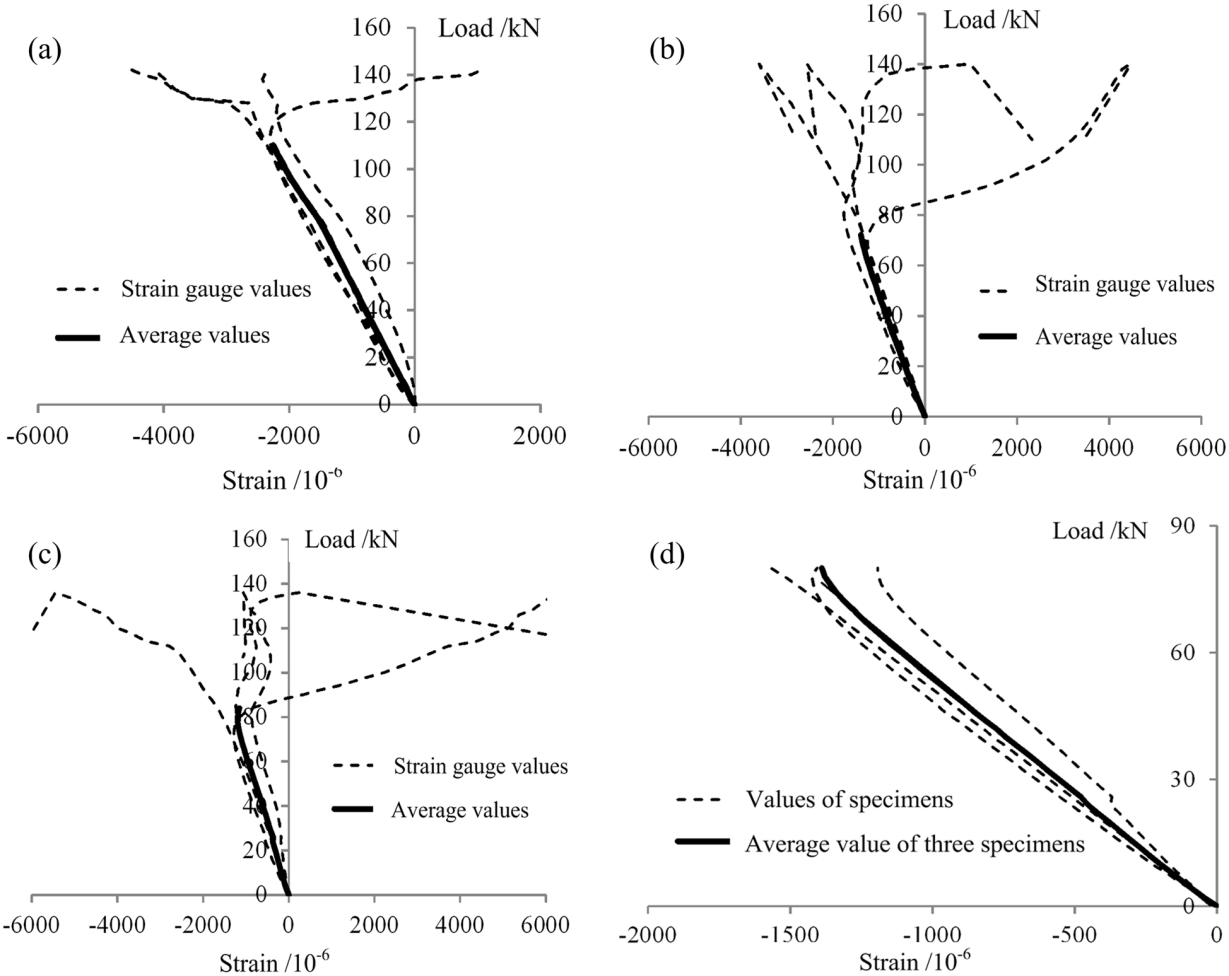
Load-axial compressive strain curves of the P-C specimens. (a) P-C-1. (b) P-C-2. (c) P-C-3. (d) Average values for all 3 P-C specimens during the elastic stage.

**Fig 17:**
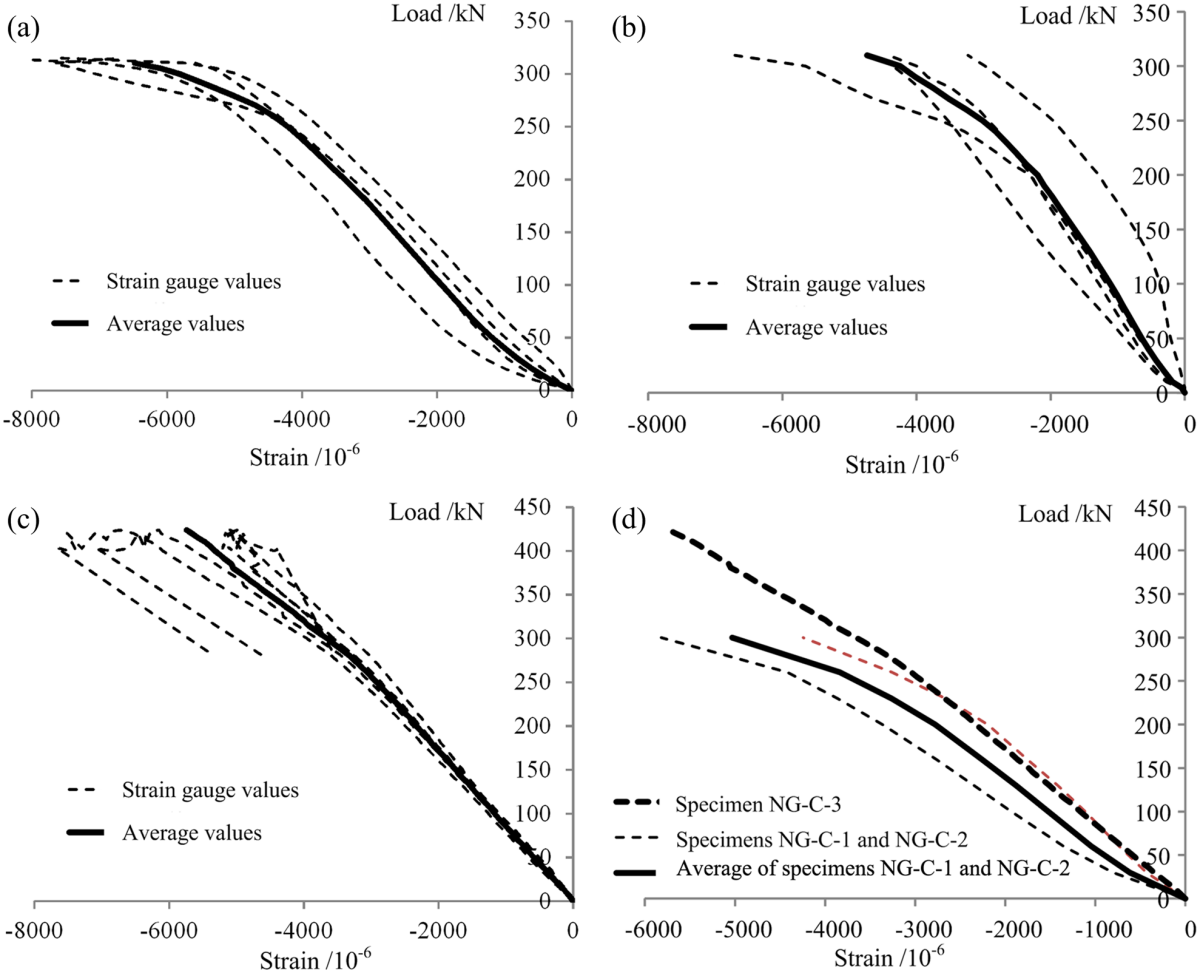
Load-axial compressive strain curves (strain on the GFRP face sheet) of the NG-C specimens. (a) NG-C-1. (b) NG-C-2. (c) NG-C-3. (d) Comparison of NG-C-3 with the other two NG-C specimens.

The load-strain curves for the three types of axial compression specimens are shown in Figs 15–17. The strains shown are the longitudinal compression strains on the central sections. For all of the W-C specimens, the curves are approximately linear peak compressive load (Fig 15). During the elastic stress stage, the maximum compression strains on specimens W-C-1, W-C-2 and W-C-3 were 0.00339, 0.00298, and 0.00331, respectively, with an average value of 0.00323 and a variable coefficient of 5.5%, indicating that the experimental results were rather stable. After the end of the elastic stage, the compressive strain on one side of each specimen continued to increase, while the strain on the other side of the specimen began to decrease until it reached zero, at which point that side of the specimen was subjected to tension. These results indicate that the specimens began to bend, which is consistent with the visual observations, as shown in Fig 13(a).

For the P-C specimens, the curves also showed a clear linear variation prior to local buckling (Fig 16). During the elastic stress stage, the maximum compressive strains on specimens P-C-1, P-C-2 and P-C-3 were 0.00235, 0.00142 and 0.00119, respectively, with an average value of 0.00162 and a variable coefficient of 28.2%, indicating that the components of this type exhibited rather large individual differences. The primary cause of this broad variation was that these were hollow thin-walled components that suffered from a variety of initial defects originating from the pultrusion process [7–9]. These initial defects had a significant impact on the distribution of internal force and, as a result, influenced the bearing capacities and failure modes of the specimens.

For the PGWC profile specimens, the initial stage of each curve exhibited obvious linear characteristics. With a continuing increase in load, the curve gradually entered a non-linear regime. There was no apparent boundary between the linear and non-linear regions, indicating that during the loading process, shift in the stress between the wood core and the GFRP sheet occurred, namely, stress redistribution, which resulted in fuller utilization of the mechanical characteristics of the wood and GFRP in the NG-C specimens. In addition, Fig 17 indicates that large stress differences between measuring points existed in the central sections of specimens NG-C-1 and NG-C-2, whereas the stress differences between measuring points in the central section of specimen NG-C-3 were relatively small. This phenomenon corresponded to their respective failure modes. The failures of the first two specimens consisted of the outward buckling failure of the GFRP sheet, whereas the latter exhibited end-crushing failure. The former result may have occurred because of poor connection at the interface between the GFRP sheet and the wood core at one or more locations, which resulted in the separation of the GFRP sheet from the wood core and caused outward buckling failure. In contrast, the connection at the interface between the GFRP sheet and the wood core in the latter specimen was good. Therefore, the stress was evenly distributed over the entire section, resulting in much later separation of the GFRP sheet from the wood core.

#### Axial compressive stress

**Fig 18:**
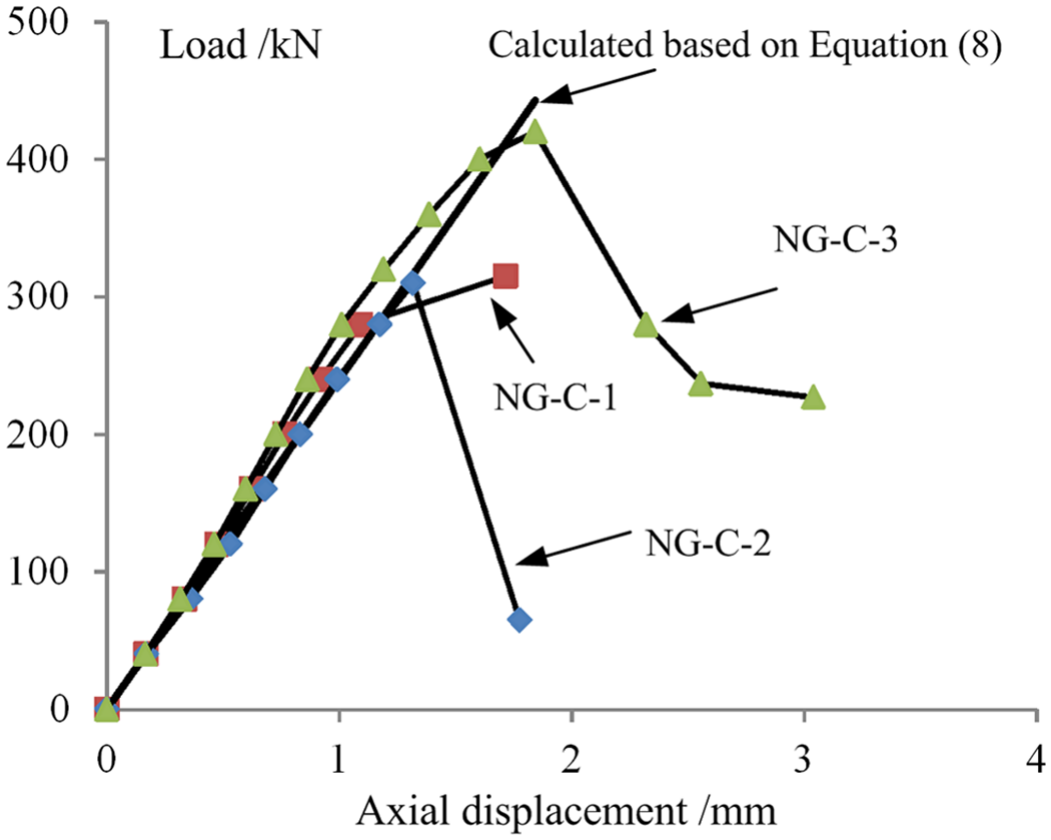
Comparison of the load-displacement curves (calculated based on Eq (8)).

Eq (8) was used to solve for the load-displacement relationship of the axial compression specimens of the NG-C type, as shown in Fig 18. Based on the results of the elastic compressive rigidity analysis presented above, the combination coefficient *φ* was taken to be 0.2. The calculated results were in good agreement with the experimental results. In a previous study conducted by Wang et al. [26], when calculating the ultimate bearing capacities of hollow sandwich columns, the authors considered the restraining effect of the FRP skins on the wood cores as well as the synthetic influence coefficient. However, no theoretically calculated results for the load-displacement relationship were given. In addition, the column cross-section in that study was circular. Therefore, the outer GFRP had a considerable constraining effect on the inner wood core. However, in another study, for a rectangular cross-sectional column, the outer GFRP was not found to have a significant constraining effect on the inner core [39]. Therefore, the combination coefficient *φ* reported here includes this constraining effect but is also related to other factors. Further study is required to identify the other influencing factors and methods of determining their values.

## Conclusions

Unsaturated polyester resin, alkali-free glass fibers, and paulownia wood were used as raw materials in the pultrusion process to manufacture the PGWC profiles investigated in this study. Three-point bending tests and axial compression tests were conducted on the PGWC profiles, and the results were compared with the results for specimens of the paulownia wood core material and specimens of hollow pultruded GFRP hollow profiles to obtain the basic bending and axial compression properties of this new composite component. The conclusions are as follows:

1. A new type of GFRP-light wood composite component is proposed in this study. This composite component consists of an inner paulownia wood core material and an adjacent GFRP face sheet and is fabricated into a continuous overall shape via the pultrusion process.
2. Under a transverse load, the proposed PGWC profiles exhibit good flexural behavior. Their bending failure modes are different from the individual bending failure modes of the paulownia wood core and the hollow GFRP profile. For a PGWC component with a non-grooved core, the failure mode corresponds to breaking failure on the pressure side of the GFRP sheet. The bearing capacity of the PGWC profile is 17.4 and 4.1 times the individual values for the paulownia wood core and the hollow pultruded GFRP profile, respectively, and 3.3 times the sum of the latter two values. Its flexural rigidity is 12.8 and 1.7 times the individual values for the paulownia wood core and the hollow pultruded GFRP profile, respectively, and 1.5 times the sum of the latter two values.
3. Under an axial compression load, the PGWC profile exhibits two failure modes, both different from the failure modes of the paulownia wood core and the hollow GFRP profile, i.e., crushing damage at the end of the GFRP and outward buckling damage after the debonding of the GFRP from the wood core; the bearing capacity of a specimen that exhibits the former failure mode is larger than the bearing capacity of one that undergoes the latter. The maximum axial compressive capacity is 4.39 and 3.03 times the individual values for the paulownia wood core and the hollow pultruded GFRP profile, respectively, and 1.79 times the sum of the latter two values. The axial elastic compressive rigidity is 3.39 and 1.86 times the individual values for the paulownia wood core and the hollow pultruded GFRP profile, respectively, and 1.20 times the sum of the latter two values.

Through this research, we characterized the basic mechanical behavior and failure modes of PGWC profiles produced via pultrusion under transverse bending loads and axial compression loads. A comparison with the paulownia wood core material and the GFRP hollow profile demonstrated that in the PGWC profiles, there exists a combined effect between the paulownia wood core material and the GFRP, which can be described by a combination coefficient determined through a mechanical analysis of the axial compression behavior. This research can thus serve as a reference for further research on the manufacturing process for GFRP composite components and their mechanical properties.

This research has certain limitations. The combination coefficient between the GFRP and the wood is an important parameter for measuring the properties of the composite component investigated in this research. Determining the value of this combination coefficient was the foundation on which the PGWC profile was designed. The project involved the study of only one cross-sectional configuration and only one type of light wood core for the PGWC profiles, and it did not address other factors that may influence the combination coefficient or methods of determining these factors, which can be considered in future studies.
